# Ultrasonographic Diagnosis of Odontogenic Cutaneous Fistula: A Case Report Demonstrating the Value of Multimodal Imaging Diagnostics

**DOI:** 10.1055/a-2618-1777

**Published:** 2025-06-16

**Authors:** Chengcheng Yu, Linlin Ruan, Wei Zhang, Hao Wang

**Affiliations:** 1 611300 Department of Ultrasound, Shandong Second Provincial General Hospital, Jinan, China; 2 611300 Department of Medical Imaging Center, Shandong Second Provincial General Hospital, Jinan, China

## Background

An oral cutaneous fistula is an external conduit that connects the oral cavity to the skin, creating an easier pathway for infections. Odontogenic cutaneous fistulas (OCFs) account for approximately 80% of oral cutaneous fistulas (Kishore Kumar RV et al. J Maxillofac Oral Surg 2012; 11: 411–415). Dental infections are a common contributing factor to morbidity (Figaro N et al. Case Rep Med 2018; 2018: 3710857). Despite the well-established causative mechanisms, clinical misdiagnosis remains at 15–20%. Clinical data indicate an 18.7% misdiagnosis rate when patients first consult dental or oral medicine professionals (Alaeddini M et al. Journal of Endodontics, 47(8): 1234–1239), primarily attributable to their insidious clinical presentation and inadequate imaging evaluation. OCFs are mostly diagnosed using X-ray and CBCT examinations. Such cases are rarely reported by ultrasound, yet its diagnostic value deserves recognition. Here, we report a case of an OCF due to misdiagnosis, which ultimately was diagnosed using ultrasound.

## Case presentation

**Fig. 1:**
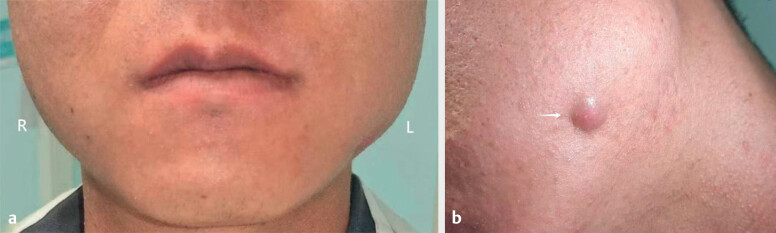
**a** : Physical examination revealed noticeable facial asymmetry, with the left side more prominent than the right. **b** : A purplish-red subcutaneous nodule measuring 1.5×1.5 cm was observed in the left mandibular angle region.

**Fig. 2:**
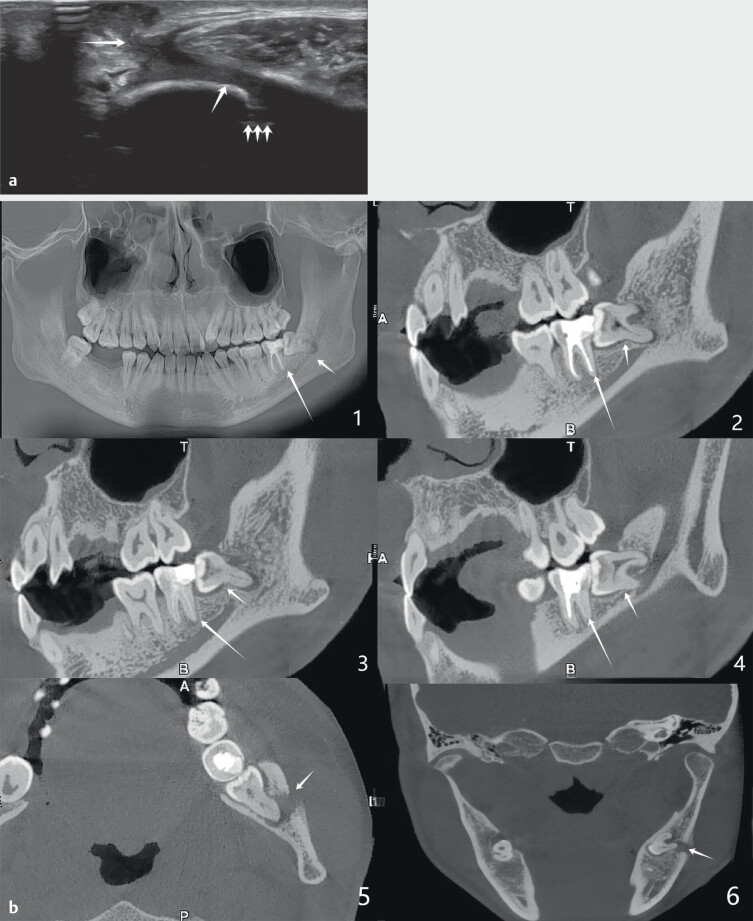
**a** : Ultrasonography revealed a hypoechoic tract extending from the skin surface through the mandibular cortex. Long arrow: Hypoechoic sinus tract; short arrow: Bone detached from the defect. **b** : CBCT imaging showed restoration of tooth #37, horizontal impaction of tooth #38, and osteolytic destruction at the root apex. 1: panoramic view; 2–4: sagittal view; 5: axial view; 6: coronal view.

A 27-year-old male patient presented to our dermatology department with a painless swelling in the left mandibular region. One month prior, he had undergone dental restoration for caries (#37) at an external dental clinic. The patient denied any history of systemic diseases, smoking, alcohol consumption, or drug allergies. Physical examination revealed a left-sided swelling of the face ( Fig. 1a ). A purplish-red subcutaneous nodule was observed in the left mandibular angle region ( Fig. 1b ), accompanied by intermittent serous discharge, which was diagnosed as a sebaceous cyst. An ultrasound examination showed a hypoechoic tract extending from the skin surface through the mandibular cortex ( Fig. 2a ). Color Doppler flow imaging (CDFI) demonstrated abundant blood flow signals around the fistula and the formation of an internal abscess cavity. These ultrasound findings were inconsistent with the acoustic image of the sebaceous cyst. The wall of the sebaceous cyst was thicker, with clearer demarcation from the surrounding skin. Generally, there is no blood flow signal in the cystic cavity and the bones are rarely eroded. Subsequent CBCT imaging showed the restoration of tooth #37, horizontal impacted-impaction of tooth #38, and osteolytic destruction of the root apex ( Fig. 2b ).

**Fig. 3:**
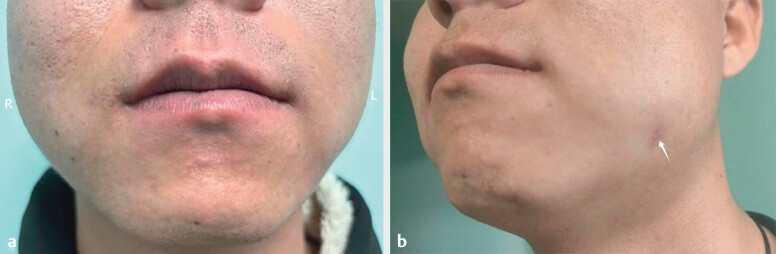
**a** : The patient's face is basically symmetrical. **b** : Resolution of the nodule with post-inflammatory hyperpigmentation at the lesion site.

**Fig. 4:**
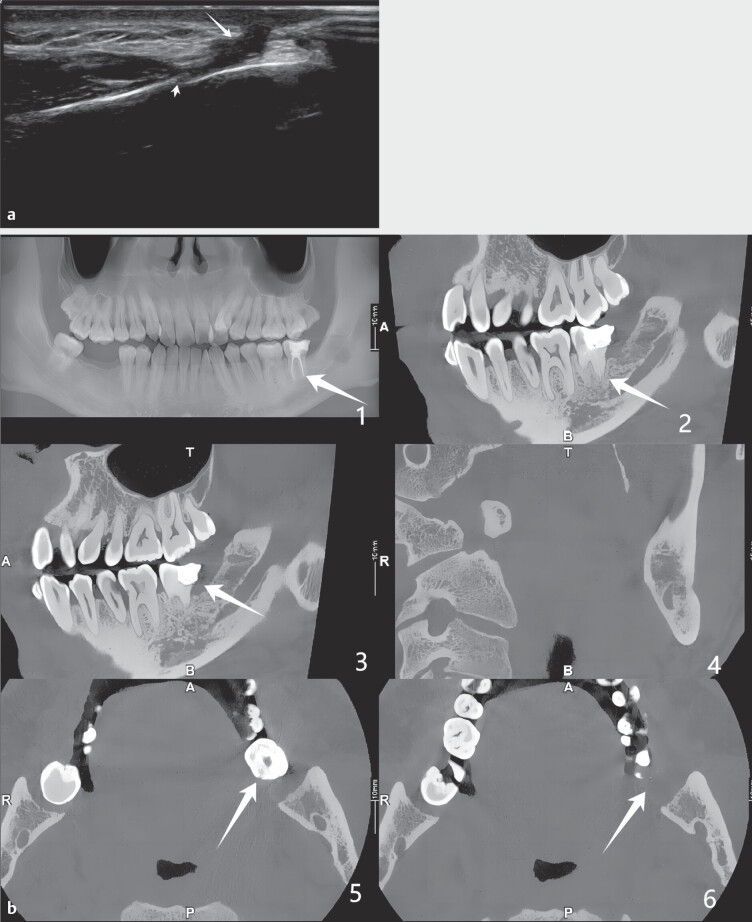
**a** : Ultrasound revealed a reduction in the hypoechoic ductal area at the site of the original nodule. Long arrow: The restored sinus tract; the restored bone. **b** : CBCT shows bone regeneration in the defect area. 1: panoramic view; 2–3: sagittal view; 4: coronal view; 5–6: axial view.

Three months later, the patient underwent treatment at a local oral surgery center. Preoperative empirical antibiotic therapy of amoxicillin–clavulanate (1 g bid for 5 days) was administered. Surgical intervention included extraction of the impacted tooth #38, debridement of the granulation tissue, fistulectomy, and closure of the internal opening. Postoperative care involved daily rinsing with 0.12% chlorhexidine mouthwash (bid for 2 weeks). The patient returned for follow-up at our hospital 10 months later. Physical examination showed that the two sides of the patient’s face were symmetrical ( Fig. 3a ) and the nodule on the left side was significantly reduced ( Fig. 3b ). Ultrasound images showed a reduction in the hypoechoic ductal area at the site of the original nodule ( Fig. 4a ), CDFI indicated a decrease in blood flow signals and resolved abscess cavity, and CBCT showed bone regeneration in the defect area ( Fig. 4b ).

## Conclusion

OCF, also known as a dental sinus tract, originates due to chronic apical periodontitis. It develops via pulp degeneration and abscess formation, leading to the discharge of purulent exudate into the gingiva and surrounding tissues. Persistent apical inflammation promotes osteolysis, which facilitates the formation of a sinus tract. The abscess may drain through the alveolar bone, anatomical spaces (such as the maxillary sinus), or the skin surface. When pus from a pulpal infection spreads from the root apex to the facial skin, a cutaneous fistula may develop, sometimes presenting as an erythema rather than a painless nodule (Guevara-Gutiérrez E et al. Int J Dermatol 2015; 54: 50–55; Kelly MS et al. BMJ Case Rep 2021; 3: 16,14; Sodnom-Ish B et al. J Korean Assoc Oral Maxillofac Surg 2021; 47: 51–56).

Most literature emphasize the treatment of the disease and the analysis of common misdiagnoses. Based on the analysis of this case, the reasons for misdiagnosis are as follows: 1. The patient's toothache symptoms were not prominent. OCFs typically present as facial erythematous nodules accompanied by periodic purulent discharge, where only 50% of cases exhibit noticeable toothache symptoms (Gao QC et al. Chinese Journal of Oral Implantina 2024; 29: 82–86). As demonstrated in this case, the patient had no symptoms of toothache and sought dermatological consultation solely due to facial swelling and nodular skin lesions. Insufficient clinical awareness of OCF manifestations and the failure to inquire in detail about recent dental treatment history led to a delayed diagnosis. 2. The insidious nature of mandibular infections. The literature indicates that 80% of OCF cases originate from mandibular infections, while only 20% arise from maxillary infections (Liu B. Chinese Journal of Leprosy Dermatology 2021; 37: 312–313). Pus from maxillary infections tends to drain into the oral vestibule due to gravity, making it easier to detect during early oral examinations. In contrast, mandibular infections often drain through subcortical bone perforation, forming subcutaneous sinus tracts in an anti-gravity direction. This makes it difficult to identify the mandibular source during clinical palpation, thereby increasing the challenge of tracing the origin of dental abscesses. 3. Limited medical resources and lack of necessary diagnostic equipment. As seen in the primary hospital where the patient was first examined, even with a complete clinical history, the absence of dental X-ray equipment prevented the early detection of the odontogenic lesion.

Oral CBCT examination allows noninvasive detailed assessment of the oral and maxillofacial skeletal structures and is commonly used by stomatologists to examine teeth. However, it has limitations in the display of the surrounding soft tissue. Just as the skin, the internal soft tissue structure was not visible on the CBCT image of this case. Ultrasound has the advantage of being able to non-invasively assess the soft tissues of a facial lesion, providing significant clinical diagnostic value. In our case, ultrasound with Doppler effectively diagnosed an odontogenic cutaneous fistula may also help with. The fistula and the damaged bone tissue were clearly visualized, and the accuracy of the ultrasound examination was further corroborated during clinical treatment.

In conclusion, we report a case of OCF diagnosed using ultrasound, demonstrating that ultrasound can serve as a first-line diagnostic modality for this condition. We urge clinical departments to incorporate ultrasound into the routine evaluation of maxillofacial masses. This approach can break down professional barriers and minimize unnecessary misdiagnoses and missed diagnoses.

